# Deep learning algorithms enable accurate identification of cassava varieties (*Manihot esculenta* Crantz) using image analysis

**DOI:** 10.3389/fpls.2026.1888478

**Published:** 2026-08-18

**Authors:** Moses Malibiche, Jonas Nickas, Hemed Msofe, James P. Legg

**Affiliations:** 1 International Institute of Tropical Agriculture (IITA), Dar es Salaam, Tanzania; 2 Tanzania Official Seed Certification Institute (TOSCI), Morogoro, Tanzania

**Keywords:** artificial intelligence, convolutional neural network, machine learning, seed systems, transfer learning, true-to-typeness

## Abstract

Deep learning is an emerging, powerful technology that facilitates object detection in plant science, enabling applications such as disease diagnostics and identification of plant species and crop varieties using image analysis. In this study, we demonstrated the success of deep learning algorithms when used in identifying Tanzania-released cassava varieties (*TARICASS4*, *Kiroba*, and *Mkuranga1*) through image analysis. Three deep learning algorithms (YOLO v8, SSD-MobileNet v2, and Faster R-CNN with ResNet-50) were trained using a dataset of 19,800 images from leaves, petioles and stems of cassava plants of the three varieties. Trained algorithms achieved promising performance in identifying the cassava varieties using each of the three plant parts (mAP50≥85.7%), with the highest values recorded for leaves (mAP50≥92.7%). Of the three models, the YOLO v8 model showed the consistent promising performance for the three plant parts, with an mAP50 of 97.3% for leaves. Further findings from confusion matrices confirmed the superior performance of YOLO v8 for leaves, with performance of ≥98% for each of the three varieties. YOLO v8 also achieved high levels of performance for stems and petioles for all varieties. Our results confirm that deep learning models can be effectively applied for AI-based identification of field-grown cassava. The approach developed for cassava has strong potential for scaling to additional cassava varieties as well as other Vegetatively Propagated Crops, and for use by seed producers and regulators who could benefit from the use of a field-usable tool for verifying variety ‘true-to-typeness’.

## 1 Introduction

Cassava (*Manihot esculenta* Crantz) is a starch-rich tuberous root crop belonging to the *Euphorbiaceae* family, feeding over 800 million people worldwide (Ding et al., 2025; Scaria et al., 2024). It is commonly propagated through vegetative plant parts such as stem cuttings or meristematic tissues (Krochmal, 1969; Roca et al., 1981). The crop can be commercially processed into different industrial products, *inter alia*, cassava starch, ethanol, high-quality cassava flour, and cassava-based sweeteners (glucose syrup) (Booth and Wholey, 1978; Howeler, 2004). Cassava adapts well to adverse growing conditions such as low rainfall, high temperature, and poor soils with low fertility, making it a suitable crop for resource-poor farmers in marginal areas (Hershey, 1993). Cassava nowadays is widely grown in the tropics and subtropics of Africa, Latin America, and Asia, where it has become an essential food security and economically important crop. Given this importance, efforts to generate improved varieties have been an important priority for breeding programmes of the Consultative Group on International Agriculture Research (CGIAR), working together with National Agricultural Research Systems (NARS) (Ceballos et al., 2004). An example of this collaboration is the work of the Tanzania Agricultural Research Institute (TARI), supported by the CGIAR centre – the International Institute of Tropical Agriculture (IITA) – which has successfully developed and officially released 26 improved cassava varieties (https://varietycatalogues.com/). These varieties have been made available to farming communities through a formal cassava seed system which was established in Tanzania in the 2010s (Douthwaite, 2020; Legg et al., 2022).

The process of releasing and registering new varieties into the formal seed system normally requires the undertaking of National Performance Trials (NPT), as well as Distinctness, Uniformity, and Stability (DUS) tests. The NPT is for estimating the Value for Cultivation and Use (VCU) of the new variety over the existing varieties. The DUS test as established by the International Union for the Protection of New Varieties of Plants (UPOV) is primarily for protecting and promoting new crop varieties (Button, 2006; Helaly et al., 2020). The DUS is conducted using the developed descriptors to confirm the unique set of traits exhibited by the new variety. These traits are typically morphological plant features. In the case of cassava, the varieties can be identified based on morphological features such as shape and colour of leaves, petioles, stems, and roots, branching type, shape of plant, as well as the presence/absence of pubescence on apical leaves (Asare et al., 2011; Ha et al., 2016). Later in the seed certification process, the confirmed DUS traits are also used to verify the true-to-typeness of the variety cultivated during seed production. The true-to-typeness is therefore hereafter referred to as the degree to which the plants of a seed lot or seed field conform to the established DUS descriptor for their declared variety. Verifying true-to-typeness can be time-consuming and prone to human errors since it may require considerable botanical expertise to distinguish the crop varieties (Helaly et al., 2020). With recent advancements in science and technology, artificial intelligence (AI) has revolutionized machine-driven object detection.

Traditionally, computer vision for object identification involved image classification models such as Support Vector Machine (SVM), logistic regression (LR), k-nearest neighbours (k-NN), naïve Bayesian, linear discriminant analysis (LDA), decision trees (DT), and random forests (RF) (Fávero et al., 2022; Wei et al., 2024). These models manually extract semantic features (hand-crafted features) for predicting or classifying the class label of the input image. This manual feature extraction compromises the accuracy and robustness of the models in diverse objects and complex backgrounds, resulting in time complexity in selecting regions (Sun et al., 2024). Deep learning (DL) algorithms address these challenges. The DL algorithms employ simple non-linear modules for hierarchical feature extraction from input images to detect and localize objects. That is, they extract the intermediate (lower level) features, which are combined and transformed into higher-level hierarchical representations of hidden patterns (subtle discriminative features) of the input dataset (Affonso et al., 2017). This hierarchical feature extraction is attributed to the intricate neural network architecture of DL, artificial neural networks (ANN), composed of interconnected simple non-linear modules by nodes (Affonso et al., 2017; LeCun et al., 2015; Jung et al., 2021). A convolutional neural network (CNN) is a form of ANN composed of convolution, pooling, and fully connected layers with the ability to identify the hidden pattern of an input dataset with grid patterns (Abdolrasol et al., 2021). This capability is mainly attributed to its convolutional layer, which extracts functionally important spatial and morphological features from image datasets.

The CNN models, as machine learning algorithms, are soft coded such that they can learn from input data without being explicitly programmed, which enables them to automatically detect and localize the object. Training the CNN models from scratch, however, requires substantially large, labelled datasets and computation resources. The transfer learning approach addresses this challenge by making feasible use of pre-trained CNN models on large benchmark datasets and fine-tuning them on a specific new smaller dataset. This reduces data requirements and time for training the models to a new specific dataset (Du et al., 2026; Plested et al., 2026; Zhu et al., 2019), thereby enabling the practical uses of CNN-based object recognition in various disciplines. Transfer learning has been extensively applied in plant science to support accurate and timely plant phenotyping and disease diagnostics through image analysis tools (Arya et al., 2022; Barbedo, 2018; Gomez et al., 2024; Jackulin and Murugavalli, 2022; James and Bradshaw, 2020; Kai et al., 2022; Laabassi et al., 2021; Murphy et al., 2024; Sun et al., 2017).

In cassava, transfer learning first enabled the utilization of CNN models in disease diagnostics for cassava brown streak disease (CBSD), cassava mosaic disease (CMD), and cassava green mite (CGM) damage (Ramcharan et al., 2017), and consequently resulted in the deployment of the AI model into a smartphone AI-driven mobile app – the ‘PlantVillage Nuru’ app (Mrisho et al., 2020; Ramcharan et al., 2019). The app has been widely used by cassava farmers across Africa in cassava disease diagnostics (Adjéi et al., 2024; Ahoya et al., 2024). Previous studies have employed CNN models to identify crop varieties using a high-throughput image analysis approach (Akbarpour et al., 2026; Bagherpour, 2026; Shynybay et al., 2026; Zhang et al., 2026). However, there is limited information on AI-based pipelines for cassava variety identification. Moreover, the majority of the previous AI studies on crop variety identification involved image acquisition in controlled settings, primarily focusing on seeds (or grains) (Akbarpour et al., 2026; Asefa et al., 2023; Bagherpour, 2026; Yang et al., 2021), or tubers (Lange et al., 2018; Mercurio and Hernandez, 2019; Shynybay et al., 2026) or whole canopies (Zhang et al., 2026). Consequently, the application of these approaches to routine field-based seed certification for field crops such as cassava remains uncertain. In the present study, we therefore hypothesized that CNN-based object detection models can distinguish cassava varieties using morphological image features from leaves, petioles, and stems, with performance varying by plant part and architecture under field conditions. To test this hypothesis, we employed transfer learning to assess the performance of CNN models in identifying cassava varieties from field-acquired images of leaves, petioles and stems. This study’s findings provide the baseline information for building the AI-pipeline for cassava variety identification in the seed certification process in the formal seed systems.

## 2 Materials and methods

### 2.1 Plant materials and acquisition of image dataset

**Figure 1:**
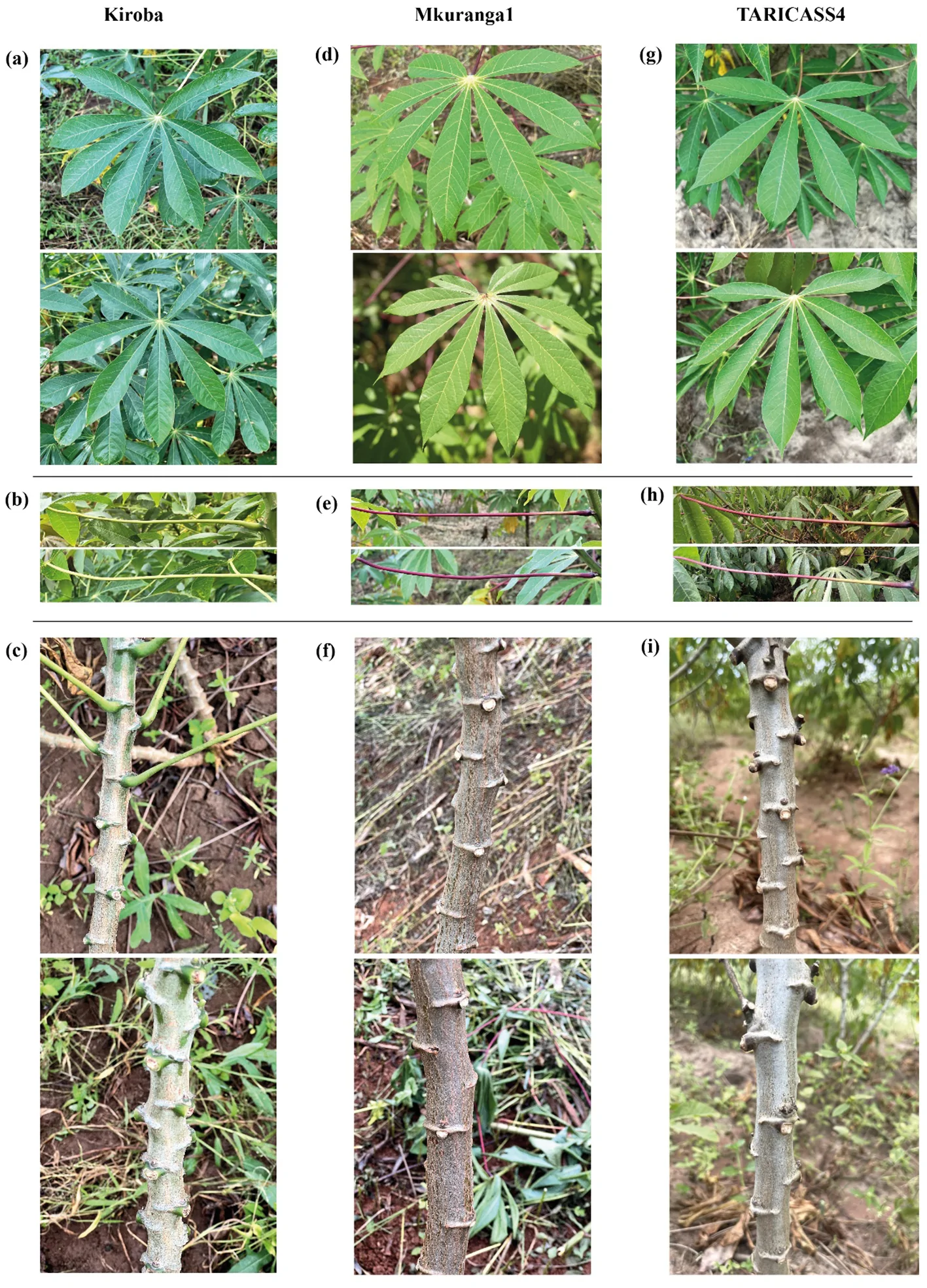
Sample of images from the collected image dataset presenting leaves, petioles, and stems of **(a–c)** *Kiroba*, **(d–f)** *Mkuranga1*, and **(g–i)** *TARICASS4* varieties.

Three cassava varieties registered and released in the formal cassava seed system of Tanzania (*TARICASS4*, *Mkuranga1*, and *Kiroba*) were selected for the study. We selected these varieties as they are widely grown in certified seed fields. *Kiroba and Mkuranga1* constituted a large nationwide share of certified seeds, 29.8% and 24.8%, respectively, in circulation by 2021 (Msami et al., 2025). The newer variety *TARICASS4* is gaining a large share in the cassava seed system of Tanzania, recording a 6.4% nationwide seed share within 1 year of release by 2021 (Msami et al., 2025). Furthermore, *Mkuranga1* and *TARICASS4* are morphologically similar but distinct from *Kiroba* (**Figure 1**). This would therefore enable us to demonstrate the feasibility of using CNN models to distinguish varieties that are morphologically similar as well as distinct. Image datasets of individual plant parts (leaf, stem and petiole) were captured using a digital camera (Nikon D3200) for each variety. The datasets were collected from the respective agroecological zones in which the selected varieties were formally released and registered for cultivation based on DUS and NPT evaluations. Specifically, datasets for *Mkuranga1* and *Kiroba* were collected in the coastal zone of Tanzania, while those for *TARICASS4* were collected in the lake zone of Tanzania. The images were acquired from an approximately 1-hectare seed field of each variety, established at a spacing of 1 meter ×1 meter which is equivalent to 10,000 plants. A total of 19,800 images were captured across the three varieties – 6,600 images for each variety (2,200 images for each plant part per variety). The plants were selected systematically to capture in-field variability, and each plant was imaged once on the leaf, petiole and stem. The selected fields contained healthy cassava plants that were 6 months old, a growth stage during which the cassava plants are easy to sample and have a good number of fully formed leaves as well as other fully expressed vegetative features. The cassava plant is in the middle of its cultivated growth cycle, and leaves are less likely to be affected by the effects of senescence or disease or pest damage.

### 2.2 Preparation of image datasets for model training

**Figure 2:**
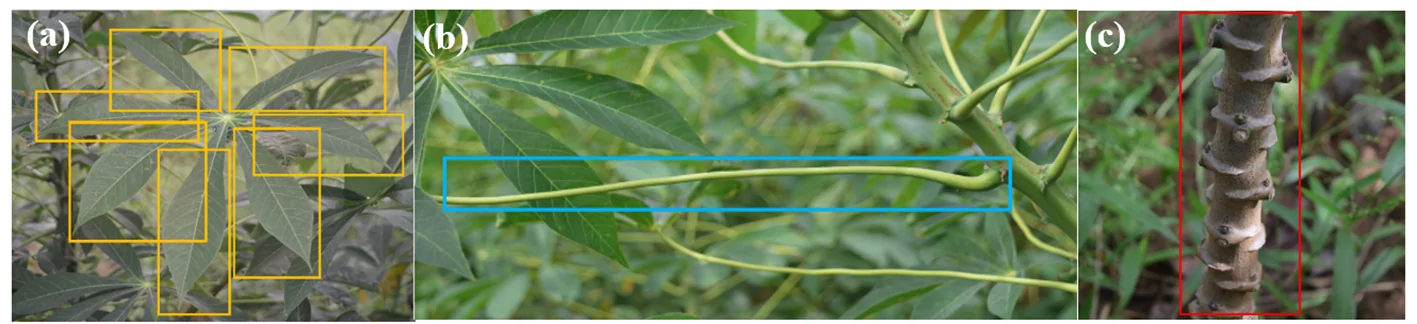
Annotated image of **(A)** leaf, **(B)** petiole and **(C)** stem during data processing.

Before training, the images were resized to 480 x 480 pixels for training the CNN models. The images were annotated using the open-source web-based Computer Vision Annotation Tool (CVAT) (https://www.cvat.ai/) (**Figure 2**). The bounding boxes were drawn on the leaf, stem and petiole per variety using CVAT (for instance *Kiroba_leaf* or *Mkuranga1_petiole*). The annotated image datasets were stored in XML and TXT files.

**Figure 3:**
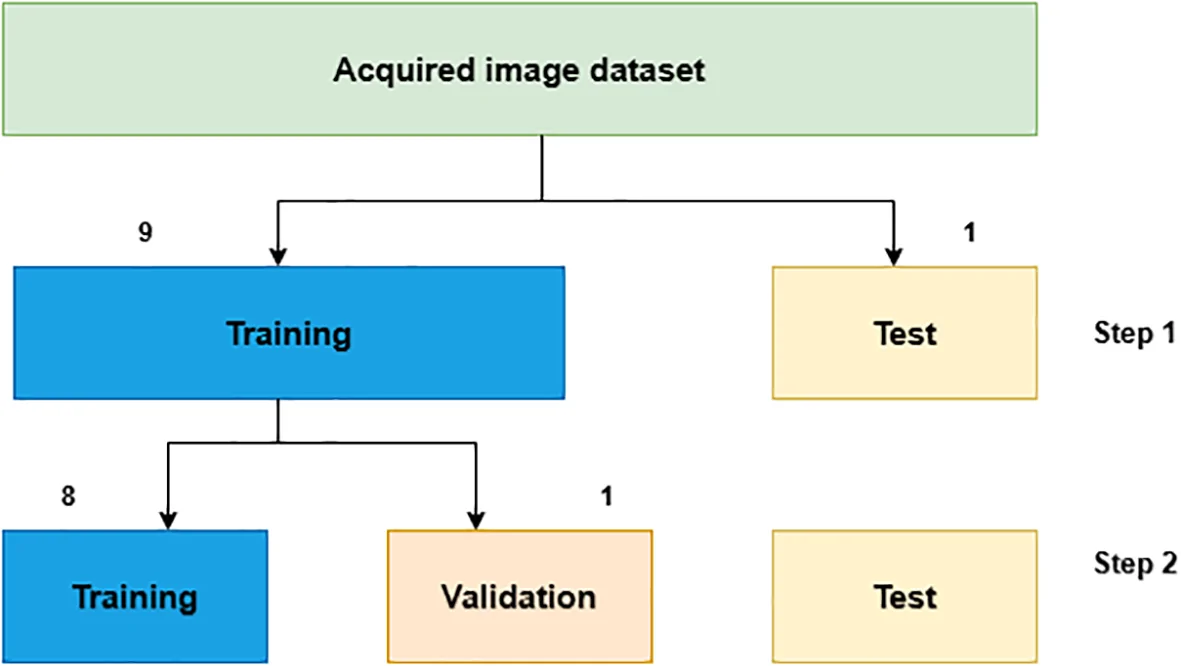
Schematic diagram describing step 1: for the initial dividing of the dataset into training and test datasets with a 9:1 ratio for model evaluation and step 2: for sub-dividing the original training dataset into training and validation datasets using an 8:1 ratio for model construction. The overall split was 80% training, 10% validation, and 10% test dataset.

After annotation, the image dataset for each variety on each plant part was initially divided into training and test datasets with a sample size ratio of 9:1 for the two datasets (90% training: 10% test) (**Figure 3**). The partition was performed at plant level such that images from a given plant were assigned exclusively to either the training or the test subset. The initial training datasets were further subdivided into training and validation datasets using an 8:1 sample size ratio (80% training: 10% validation) (**Figure 3**). This is equivalent to an overall ratio of 8:1:1 (80% training: 10% validation: 10% test dataset). This was applied to the 2,200 images for each variety per plant part, yielding 1,760 training images, 220 validation, and 220 test images.

### 2.3 Model training

The transfer learning approach was used to train the pre-trained CNN models. In contrast to the conventional approaches, where model training was done using time-consuming manual feature extraction, the CNNs learn features automatically from the training data, enabling them to carry out tasks such as object detection and image classification. The transfer learning approach is used to speed up the training process by taking a fully pre-trained model and retraining it for the new datasets (Gomez et al., 2024; Mohanty et al., 2016; Ramcharan et al., 2017). Therefore, we conducted the training using three different state-of-the-art pre-trained CNN architectures with a good balance of speed and accuracy: Single Shot MultiBox Detector (SSD) with MobileNet v2, YOLO v8 (You Only Look Once) and Faster Regional-CNN (Faster R-CNN) with ResNet-50 (hereafter referred to as Faster R-CNN ResNet-50). The YOLO v8 small size variant of YOLO v8 was used. The three models were selected because they are widely adopted and highly influential object detection architectures that represent different trade-offs between accuracy, computational complexity, inference speed, and model deployment suitability. These models have demonstrated strong performance in object detection applications and serve as representative benchmarks of contemporary detection approaches. SSD-MobileNet v2 was chosen as a lightweight architecture optimized for mobile and edge-device deployment, YOLO v8 was selected as a modern state-of-the-art one-stage detector that provides an excellent balance between accuracy and speed, and Faster R-CNN ResNet-50 was included as a well-established two-stage detector known for strong detection performance. Using these three models we get a comprehensive evaluation of accuracy-efficiency trade-offs for cassava variety identification while considering the ultimate goal of smartphone-based deployment of the model. The backbones of these models were initialized with weights pretrained on the COCO dataset (Common Objects in Context) containing 1.5 million labelled object instances. COCO, however, comprises only generic object-categories with no cassava morphological features. These generic features were adapted to cassava datasets by fine-tuning the full network (backbone and detection heads) on the cassava image datasets. Instead of freezing any layers, all layers were left trainable to allow each model to fully adapt its weights to the morphological features that distinguish cassava varieties. The SSD-MobileNet v2 presents the architecture in which the SSD is integrated with MobileNet v2. MobileNet v2 is a lightweight architecture designed for mobile applications and acts as a backbone for SSD by extracting features of the object from an input image dataset that can be detected quickly by the SSD and at higher accuracy (Palwankar and Kothari, 2022). Also, Faster R-CNN ResNet-50 is the architecture with ResNet-50 as the backbone for feature extraction, so that features can be detected quickly and accurately by Faster R-CNN (Renjun et al., 2022). The selected CNN-based object detection models were implemented in Python and trained for 35 epochs using deep learning frameworks (PyTorch for YOLO v8 and TensorFlow for SSD-MobileNet v2 and Faster R-CNN ResNet-50) within Jupyter Notebook on Google Colab, using a T4 GPU with 16 GB of video random access memory (VRAM) and 2,560-core CUDA parallel processor architecture.

### 2.4 Model optimization through hyperparameter tuning

To improve the model performance and efficiency, we performed model optimisation using hyperparameter tuning. Hyperparameter tuning was performed through manual experimentation, and identical augmentation settings were applied across all models to ensure a fair comparison. This was done at 0.005 learning rate, 16 batch size, 35 epochs. To improve model generalisation to new data, data augmentation was done for training data, including scale variation (0.4), random rotation (± 15^°^), brightness (0.4), saturation (0.6), and HSV hue (0.015). An ‘early stopping’ function was implemented with a patience value of 7 epochs during training to prevent overfitting. When overfitting occurs, the model memorises the training data so much that it fails to perform well on new unseen data. To avoid overfitting, we implemented the early stopping function to end the training when the performance of the model on validation data stopped improving.

### 2.5 Performance evaluation of the trained CNN model pipelines

The mean average precision (mAP) score, confusion matrix, precision and recall, and F1-score, were used to evaluate model performance on different plant parts of the three cassava varieties. The models were tested on 1,980 new images (660 for each plant part; 220 per variety per plant part).

#### 2.5.1 Mean average precision

A mean average precision at the Intersection over Union (IoU) threshold of 0.5 (mAP50) was used to measure how well the trained CNN models performed in identifying different cassava varieties. The mAP50 is commonly used as a performance metric in object detection using machine learning to estimate how accurately the model predicts the bounding boxes relative to the ground truth annotations. The mAP50 describes the correct predicted bounding box that overlaps with the ground truth box (labelled box) by at least 50% (IoU≥0.5). Additionally, we estimated the mAP over a range of IoU from 0.5 to 0.95 (mAP50-95). The mAP50 and mAP50–95 were generated from the test dataset during model evaluation.

#### 2.5.2 Confusion matrix

Confusion matrices were generated during model evaluation on the test dataset. Each predicted bounding box was matched to a ground-truth annotation when the two boxes overlapped by at least the IoU threshold of 0.5 defined in Section 2.5.1. On this basis, the detection outputs for each plant part were categorised, for each variety, as true positive (TP), false positive (FP) or false negative (FN). A TP is an annotated plant part of that variety that was detected and correctly labelled as that variety. A FP is a detection labelled as that variety that either matched an annotated plant part of a different variety, matched no annotated plant part, or duplicated a detection already matched to the same plant part. A FN is an annotated plant part of that variety that was either labelled as a different variety or not detected at all. A single misclassification therefore contributes one FN to the true variety and one FP to the predicted variety, which is why the off-diagonal cells of the matrix bear on both recall and precision. True negatives were not defined, because the number of possible background regions in an image is unbounded, and none of the metrics reported in this study uses TN.

#### 2.5.3 Precision, recall and F1-score

Precision is the proportion of detections labelled as a given variety that were correct (Equation 1), while recall is the proportion of annotated plant parts of that variety that the model correctly identified (Equation 2) (Gomez et al., 2024). Recall is also referred to as the sensitivity of the model in identifying the true class. Precision and recall together give the F1-score, a single summary measure of model performance expressed as the harmonic mean of the two (Equation 3). All three metrics were computed from the TP, FP and FN counts defined in Section 2.5.2, separately for each variety and plant part. Each detection is accompanied by a confidence score between 0 and 1 representing the model’s certainty in that prediction. The models were evaluated using the default validation settings of their respective frameworks (YOLO v8, SSD-MobileNet v2, and Faster R-CNN) rather than a manually tuned confidence threshold. The values reported as “Average” in **Tables 1**–**3** are the unweighted means across the three varieties.

**Table 1:** The precision, recall, and F1-score values of the trained CNN models on identifying the selected varieties based on leaf.

Metrics	Varieties	Selected CNN models		
		SSD-MobileNet v2	YOLO v8	Faster R-CNN ResNet-50
mAP50	*Kiroba*	0.962	0.972	0.913
	*Mkuranga1*	0.966	0.967	0.964
	*TARICASS4*	0.963	0.978	0.903
	Average	0.964	0.973	0.927
mAP50-95	*Kiroba*	0.782	0.796	0.785
	*Mkuranga1*	0.918	0.863	0.806
	*TARICASS4*	0.839	0.936	0.815
	Average	0.846	0.865	0.801
Precision	*Kiroba*	0.928	0.936	0.872
	*Mkuranga1*	0.830	0.850	0.799
	*TARICASS4*	0.926	0.922	0.903
	Average	0.894	0.903	0.857
Recall	*Kiroba*	0.918	0.973	0.944
	*Mkuranga1*	0.998	0.978	0.897
	*TARICASS4*	0.905	0.984	0.889
	Average	0.940	0.979	0.909
F1-score	*Kiroba*	0.923	0.954	0.907
	*Mkuranga1*	0.906	0.910	0.845
	*TARICASS4*	0.915	0.952	0.896
	Average	0.915	0.940	0.882

**Table 2:** The precision, recall, and F1-score values of the trained CNN models on identifying the selected varieties based on petiole.

Metrics	Varieties	Selected CNN models		
		SSD-MobileNet v2	YOLO v8	Faster R-CNN ResNet-50
mAP50	*Kiroba*	0.839	0.832	0.804
	*Mkuranga1*	0.825	0.942	0.928
	*TARICASS4*	0.904	0.896	0.861
	Average	0.857	0.890	0.864
mAP50-95	*Kiroba*	0.554	0.549	0.680
	*Mkuranga1*	0.755	0.732	0.729
	*TARICASS4*	0.643	0.688	0.688
	Average	0.651	0.656	0.699
Precision	*Kiroba*	0.776	0.778	0.777
	*Mkuranga1*	0.803	0.871	0.795
	*TARICASS4*	0.842	0.837	0.839
	Average	0.807	0.829	0.803
Recall	*Kiroba*	0.716	0.687	0.764
	*Mkuranga1*	0.751	0.845	0.728
	*TARICASS4*	0.802	0.794	0.781
	Average	0.756	0.775	0.758
F1-score	*Kiroba*	0.745	0.730	0.770
	*Mkuranga1*	0.776	0.858	0.760
	*TARICASS4*	0.822	0.815	0.809
	Average	0.781	0.801	0.780

**Table 3:** The precision, recall, and F1-score values of the trained CNN models on identifying the selected varieties based on stem.

Metrics	Varieties	Selected CNN models		
		SSD-MobileNet v2	YOLO v8	Faster R-CNN ResNet-50
mAP50	*Kiroba*	0.889	0.902	0.938
	*Mkuranga1*	0.903	0.925	0.879
	*TARICASS4*	0.894	0.955	0.884
	Average	0.895	0.927	0.901
mAP50-95	*Kiroba*	0.804	0.892	0.922
	*Mkuranga1*	0.751	0.916	0.864
	*TARICASS4*	0.888	0.943	0.826
	Average	0.814	0.917	0.871
Precision	*Kiroba*	0.756	0.954	0.826
	*Mkuranga1*	0.724	0.662	0.661
	*TARICASS4*	0.886	0.916	0.871
	Average	0.789	0.844	0.785
Recall	*Kiroba*	0.972	0.982	0.991
	*Mkuranga1*	0.988	0.982	0.979
	*TARICASS4*	0.981	0.980	0.969
	Average	0.980	0.981	0.980
F1-score	*Kiroba*	0.850	0.968	0.901
	*Mkuranga1*	0.835	0.791	0.789
	*TARICASS4*	0.931	0.947	0.917
	Average	0.872	0.902	0.869

(1) $$Precision=\frac{True\;Positive\;(TP)}{True\;Positive\;(TP)+False\;Positive\;(FP)}$$

(2) $$Recall=\frac{True\;Positive\;(TP)}{True\;Positive\;(TP)+False\;Negative\;(FN)}$$

(3) $$F1-score=2\times \frac{Precision\;\times \;Recall}{Precision\;+\;Recall}$$

## 3 Results

### 3.1 Overall performance of the trained models in all classes (varieties)

The results recorded consistently higher mAP50 compared to mAP50–95 across all plant parts throughout the model training process (**Tables 1**–**3**). YOLO v8 attained better mAP50 and mAP50–95 values than the other two models across all plant parts, except for the petiole, where a higher mAP50-95 (69.9%) was observed for Faster R-CNN ResNet-50 compared to 65.6% for YOLO v8 (**Table 2**). However, all CNN models expressed higher mAP50 for leaf (≥92.7%) (**Table 1**). Moreover, the models exhibited higher mAP50-95 (≥81.4%) on the stem (**Table 3**), while expressing the lowest mAP50–95 scores (≤69.9%) on the petiole (**Table 2**). Moreover, YOLO v8 showed overall good trend on precision, recall and F1-score across all plant parts compared to other models (**Tables 1**–**3**). Also, all models exhibited promising precision, and F1-score values on leaf part (**Tables 1**–**3**), while the recall value was higher on stem part (**Table 3**).

**Figure 4:**
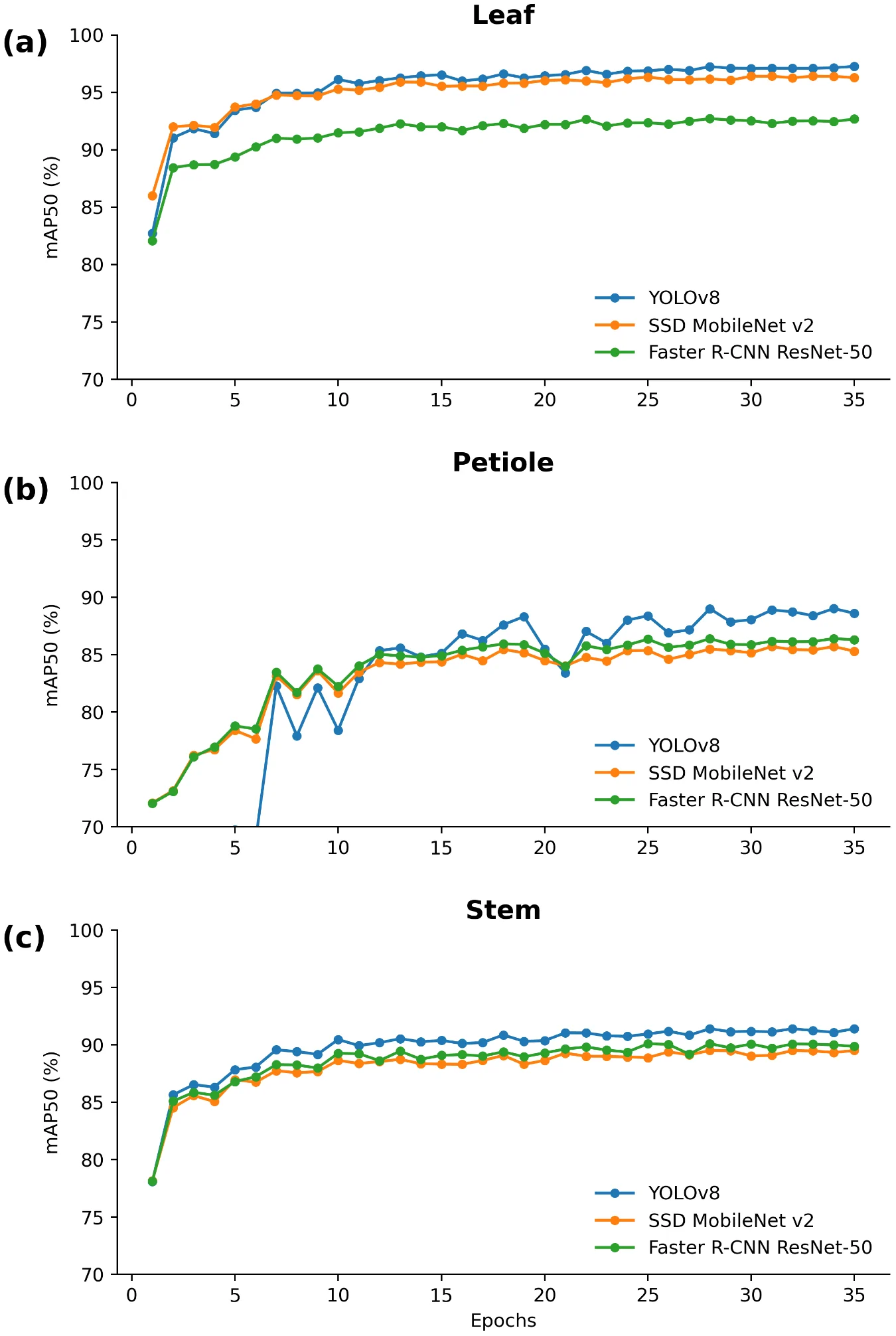
Changes in mAP50 recorded across the epochs for the three models for **(a)** leaf, **(b)** petiole, and **(c)** stem during testing.

**Figure 5:**
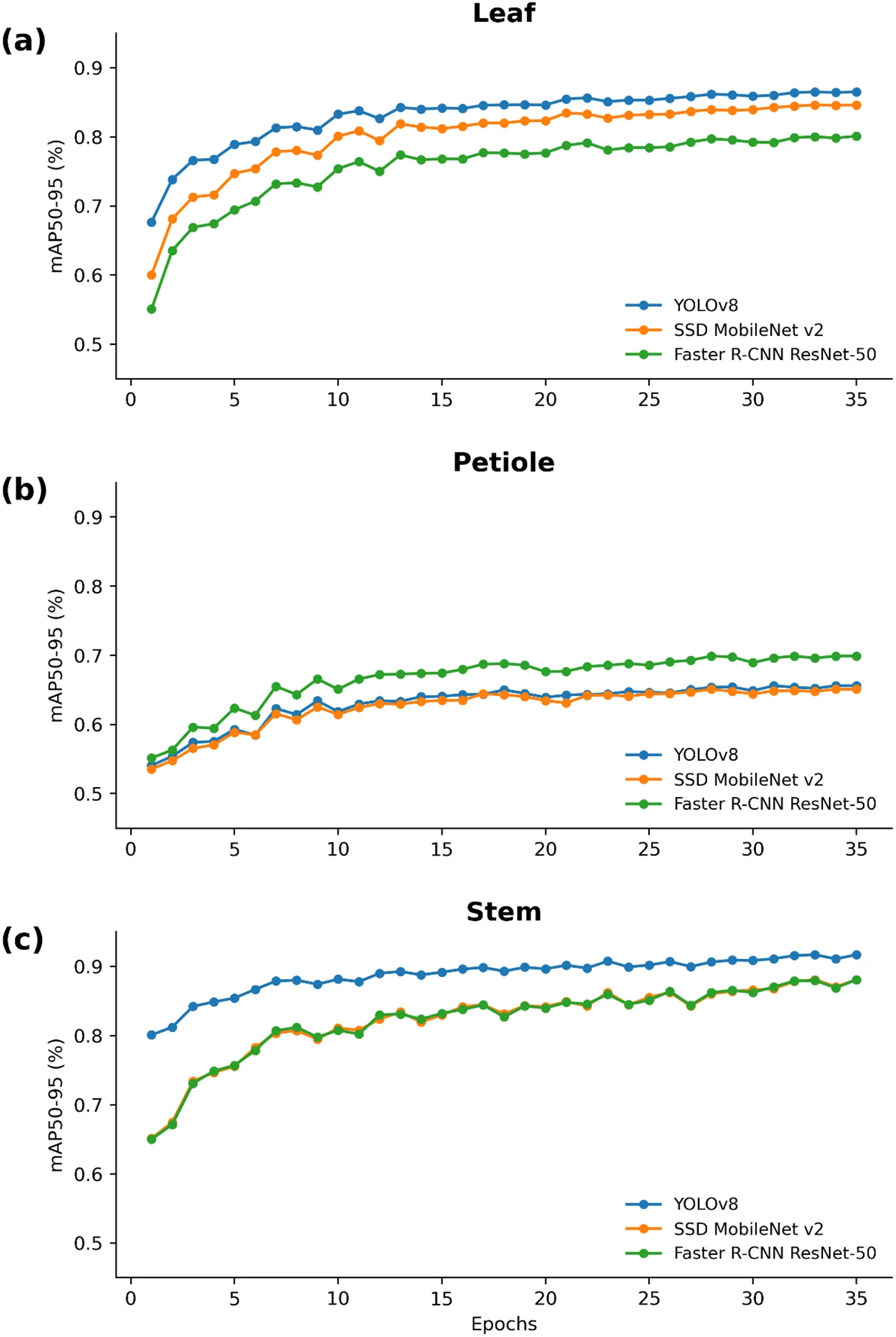
Changes in mAP50–95 recorded across epochs for the three trained models for **(a)** leaf, **(b)** petiole, and **(c)** stem.

YOLO v8 maintained a higher mAP50 than the other models for all plant parts across epochs during model evaluation (testing) (**Figure 4**). Also, it showed the progressive higher mAP50–95 curve across the epochs for all plant parts, except for petiole where Faster R-CNN ResNet-50 showed higher curve (**Figure 5**).

### 3.2 Comparative performance of the CNN models using confusion matrices

**Figure 6:**
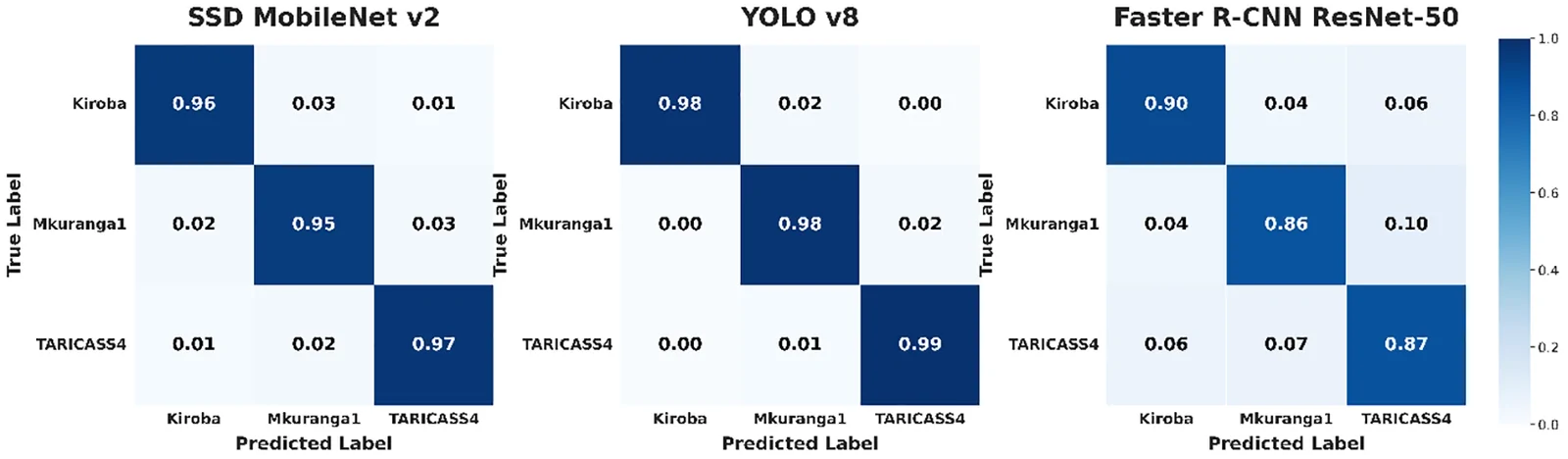
The confusion matrix describing the performance (%) of CNN models for detecting each cassava variety using leaf test dataset.

**Figure 7:**
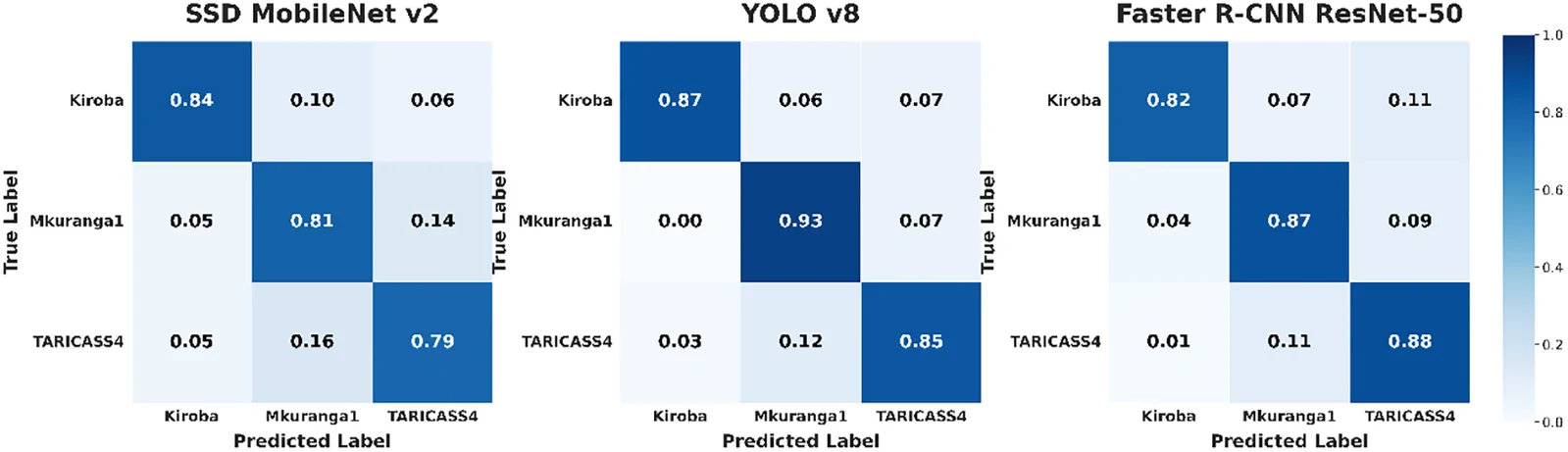
The confusion matrix describing the performance (%) of CNN models for detecting each cassava variety using petiole test dataset.

**Figure 8:**
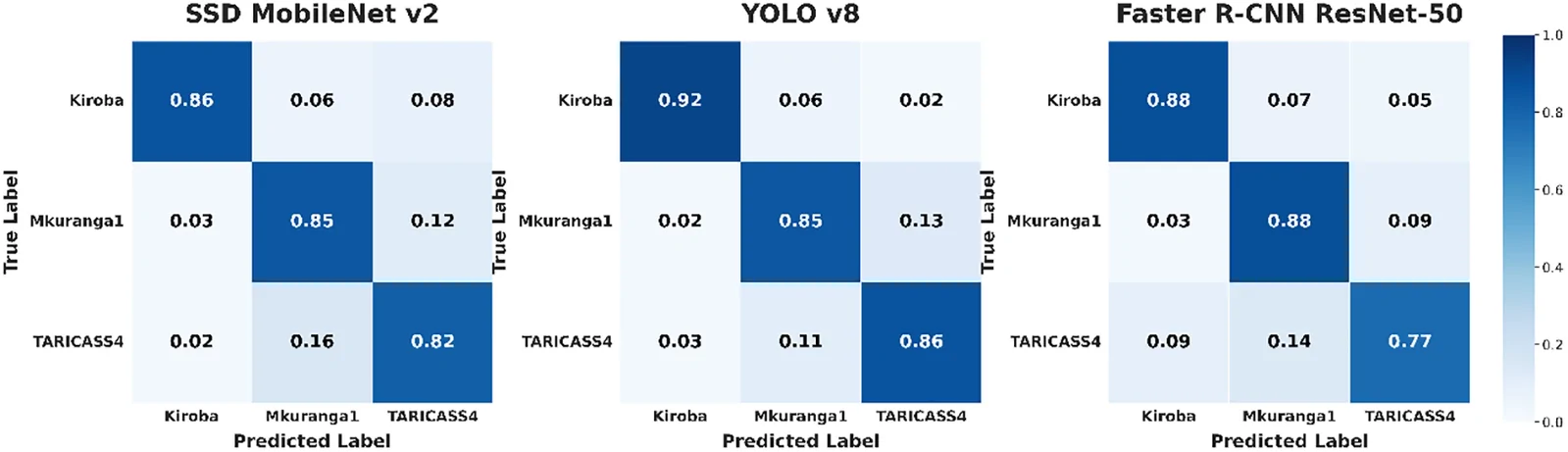
The confusion matrix describing the performance (%) of CNN models for detecting each cassava variety using stem test dataset.

The confusion matrices presenting the specific performance of each trained model for every plant part in identifying cassava varieties are shown in **Figures 6**–**8**. Based on the leaf datasets, YOLO v8 performed better, with ≥98% performance for identifying the three cassava varieties (**Figure 6**). The model identified true *TARICASS4* leaves with a performance of 99% and achieved 98% when identifying the true leaves of both *Kiroba* and *Mkuranga1* (**Figure 6**). SSD-MobileNet v2 specifically outperformed Faster R-CNN ResNet-50 in identifying the selected varieties based on leaf datasets (**Figure 6**). In predicting the true leaves of *Kiroba*, *Mkuranga1*, and *TARICASS4*, *SSD-MobileNet v2* correctly detected 96%, 95% and 97% of them, respectively, compared to 90%, 86% and 87%, respectively, achieved by Faster R-CNN ResNet-50 (**Figure 6**).

When we trained the models using the petiole dataset, YOLO v8 outperformed the other models, achieving 87% and 93% for *Kiroba* and *Mkuranga1* respectively (**Figure 7**). Considering the other two models, Faster R-CNN ResNet-50 expressed superior performance on *Mkuranga1* (87%) compared to SSD-MobileNet v2 (81%). Conversely, the opposite trend was recorded for *Kiroba* where SSD-MobileNet v2 performed better than Faster R-CNN ResNet-50 (**Figure 7**). Faster R-CNN ResNet-50 performed well (88%) for *TARICASS4* petioles followed by YOLO v8 (85%), and SSD-MobileNet v2 (76%) (**Figure 7**).

YOLO v8 gave the highest levels of performance for stems of *Kiroba* (92%) and *TARICASS4* (86%) (**Figure 8**). Additionally, Faster R-CNN ResNet-50 consistently outperformed SSD-MobileNet v2 when using stem datasets for variety identification, except for *TARICASS4* (**Figure 8**). Faster R-CNN ResNet-50 also performed slightly better (88%) for stems of *Mkuranga1* compared to YOLO v8 (85%) (**Figure 8**).

In some cases, the CNN models exhibited detection confusion on *Mkuranga1* and *TARICASS4* across all plant parts (**Figures 6**–**8**). For instance, in the leaf part, Faster R-CNN ResNet-50 misidentified 10% of *Mkuranga1* true classes as *TARICASS4* (**Figure 6**). Similarly, SSD-MobileNet v2 misidentified 16% of true class *TARICASS4* petioles as *Mkuranga1* (**Figure 7**) and 14% of true class *Mkuranga1* stems as *TARICASS4* (**Figure 8**).

## 4 Discussion

The integration of deep learning algorithms with high-throughput analysis of image-based biological data offers a powerful emerging mechanism that can rapidly and accurately extract, analyse, and interpret meaningful image patterns, enabling various smart agricultural applications such as digital disease diagnostics and plant phenomics (Gomez et al., 2024; Pound et al., 2017; Sheikh et al., 2024). The research described here presents the first demonstration of the application of deep learning algorithms in cassava variety identification using leaf, stem, and petiole-based image data. The trained algorithms were more effective in correctly identifying cassava varieties using leaf parts. The findings align with those of Du et al. (2020) and Zhang and Li (2022), who reported the notably higher performance that was achieved by deep learning algorithms in phenotypic evaluation and identification of lettuce varieties through high-throughput analysis of leaf images. The higher variability of distinct morphological features of cassava variety leaves such as the venation pattern, colour of leaf veins, leaf colour, shape of the central leaf, leaf surface texture, number of leaf lobes, length of lobes, shape of lobes, width of lobes, lobe margins, and sinuosity of lobes could be underlying factors for the noted higher accuracy in varietal identification when using leaves (Afonso et al., 2014; Kumar et al., 2025; Semman et al., 2025). More variable traits make it easier for models to learn the distinctive features among the varieties, enhancing accuracy in predicting the true labelled input data. However, the higher mAP50–95 recorded on the stem part by YOLO v8 and Faster R-CNN ResNet-50 even compared to leaf reflects their superior ability to accurately localize stem features consistently under stricter IoU thresholds. This may be attributed to stable and distinct stem features such as internode length, and stem colour important for precise object detection. The higher recall value recorded by the models on the stem part indicates the models missed few detections during the testing on stem image data. In contrast, petioles are relatively narrower and might have fewer morphological features. This may make it difficult for the model to learn and accurately localize variety-specific features. Also, the variability in angle of image capture, and occlusion for petiole may further reduce the model performance.

All the trained CNN models in this study showed promising potential for identifying cassava varieties, but our results consistently highlight YOLO v8 as the good model for accurately identifying cassava varieties in the field through image analysis. However, the finding of this study also demonstrates that performance of the model is not solely relying on architecture, but the interaction between model architecture and varietal specific morphological features. For instance, Faster R-CNN ResNet-50 performance benefited *Mkuranga1* stems meanwhile YOLO v8 provided advantage for *Kiroba* and *TARICASS4* stems. *Mkuranga1 and TARICASS4* seem to be very difficult varieties to distinguish, especially using Faster R-CNN ResNet-50 on leaf, and SSD-MobileNet v2 on both petiole and stems. These models struggled on these respective parts to separate *TARICASS4* and *Mkuranga1*, indicating the close morphological similarity between the two varieties, with *Kiroba* showing greater distinctiveness. SSD-MobileNet v2 struggled more in distinguishing these varieties than Faster R-CNN ResNet-50. This also emphasizes that model performance is not only influenced by architecture but also morphological similarities of cassava varieties.

YOLO v8, like other YOLO series (for instance, YOLO v1, YOLO v5, YOLO v7), enables object detection as a single regression problem, such that the model can predict the bounding boxes by processing the entire input image once (one pass processing) (Hussain, 2023). The architecture of YOLO v8 in particular, is primarily designed for high-speed inference, which enhances the speed and efficiency of object detection compared to the multi-stage detection algorithms such as Faster R-CNN with ResNet-50 (Khan et al., 2025). Ding et al. (2024) also reported that YOLO v8 was one of the promising object detection algorithms for high-throughput image analysis. The YOLO v8 architecture offers an opportunity for streamlined object detection by introducing the one-stage anchor-free object detection system that enhances model accuracy (He et al., 2024; Khan et al., 2025; Zhang et al., 2025). The model can perform more efficiently on data collected from diverse environments by not depending on the predefined anchor boxes to generate the bounding boxes. This suits well with the field settings where we collected the image dataset of cassava varieties. The YOLO v8 architecture has an enhanced Feature Pyramid Network (FPN) linked to a Path Aggregation Network (PAN) for merging all the features from the backbone of the architecture. This integration enhances multi-scale semantic and positional information, which consequently enhances model accuracy in detecting the subtle and complex pattern of varying size (Khan et al., 2025; Zhang et al., 2025). It justifies why YOLO v8 was less confused in distinguishing the varieties, including morphologically close ones, using image datasets collected in different settings, as compared to Faster R-CNN with ResNet-50 and SSD-MobileNet v2.

Our results demonstrated that the model achieved very high mAP50 values across all plant parts when identifying the three varieties. The higher mAP50 values achieved by YOLO v8 demonstrated how well the model identifies and localizes different parts of respective cassava varieties in a way that the predicted bounding boxes overlap for at least 50% within the area of ground truth. Therefore, the model effectively balanced its precision and recall in the accurate identification of the plant parts (leaves, petioles, and stems) of the cassava varieties used. Since YOLO v8 has demonstrated promising results across leaf, petiole, and stem, it should be possible to design a YOLO v8-powered mobile system for varietal identification using all three features. Using all three features combined may result in greater accuracy in variety identification. Also, realising the object detection capability of YOLO v8 with enhanced multi-scale semantic and positional information, it is highly likely that the model could be successfully scaled to include other varieties in the seed systems while monitoring the generalizability of the model under the field conditions with increasing number of varieties.

## 5 Conclusion

In conclusion, our study represents a critical initial step in AI-based cassava variety identification, demonstrating the feasibility of using deep learning algorithms to accurately identify cassava varieties. The study provides fundamental insights into the future use of deep learning algorithms in verifying true-to-typeness within cassava seed systems, noting good performance of YOLO v8 in cassava variety identification. The findings also highlighted the leaf as the most informative plant part for achieving the highest level of performance in variety identification. This deep learning-based variety identification approach could also be a more effective and accurate method for identifying varieties with subtle differences (hidden patterns) among varieties that cannot be easily recognized by the human eye.

## 6 Limitation of the study and future recommendations

The study is limited by training the CNN models using images collected at 6 months of planting. Hence, we recommend further research to assess the performance of the models across different growth stages. A further limitation of this study is the confounding of variety with agroecological zone, since *Kiroba* and *Mkuranga1* images were acquired in coastal zone while *TARICASS4* images were acquired in lake zone. Hence, the models could have learned to identify *TARICASS4* partly on the basis of the lake-zone-specific environmental conditions rather than morphological features alone. This represents a threat to the internal validity of variety identification, not only a limitation of its generalizability. Therefore, future research involving data acquisition across the zones is recommended. Additionally, the number of cassava varieties was limited, however, future work that adds more varieties should monitor the model’s generalizability with the added varieties.

## Data Availability

The original contributions presented in the study are included in the article/supplementary material. Further inquiries can be directed to the corresponding author.

## References

[B1] Abdolrasol M. G. M. Suhail Hussain S. M. Ustun T. S. Sarker M. R. Hannan M. A. Mohamed R. . (2021). Artificial neural networks based optimization techniques: a review. Electronics 10, 2689. doi: 10.3390/electronics10212689 30654563

[B2] Adjéi E. A. Traoré K. Sawadogo/Compaoré E. M. F. W. Amoakon W. J. L. Kouassi N. K. Kouassi M. K. . (2024). Perception and adoption by cassava farmers of the PlantVillage Nuru application disseminated in the agricultural environment of Côte d’Ivoire: a case study in the departments of Dabou, Bouaké, and Man. Front. Agron. 6. doi: 10.3389/fagro.2024.1433204

[B3] Affonso C. Rossi A. L. D. Vieira F. H. A. de Leon Ferreira A. C. P. (2017). Deep learning for biological image classification. Expert Syst. Appl. 85, 114–122. doi: 10.1016/j.eswa.2017.05.039 38826717

[B4] Afonso S. J. Ledo C. D. S. Moreira R. F. C. Silva S. D. O. Leal V. D. J. Conceição A. D. S. (2014). Selection of descriptors in a morphological characteristics considered in cassava accessions by means of multivariate techniques. J. Agric. Veterinary Sci. 7, 13–20. doi: 10.9790/2380-07151320

[B5] Ahoya D. K. D. Sawadogo-Compaore E. M. F. W. Yabi J. A. Zandjanakou-Tachin M. Houngue J. A. Houedjissin S. S. . (2024). Factors influencing adoption of the PlantVillage Nuru application for cassava mosaic disease diagnosis among farmers in Benin. Agriculture 14, 2001. doi: 10.3390/agriculture14112001 30654563

[B6] Akbarpour O. Akbari S. Fanourakis D. Taheri-garavand A. (2026). Deep learning-based seed variety classification: a case study in maize. BMC Plant Biol. 26, 105. doi: 10.1186/s12870-025-07919-3 41402752 PMC12821201

[B7] Arya S. Sandhu K. S. Singh J. Kumar S. (2022). Deep learning: as the new frontier in high-throughput plant phenotyping. Euphytica 218, 47. doi: 10.1007/s10681-022-02992-3 30311153

[B8] Asare P. A. Galyuon I. K. A. Sarfo J. K. Tetteh J. P. (2011). Morphological and molecular based diversity studies of some cassava (Manihot esculenta Crantz) germplasm in Ghana. Afr. J. Biotechnol. 10, 13900. doi: 10.5897/ajb11.929

[B9] Asefa B. G. Tsige F. Mehdi M. Kore T. Lakew A. (2023). Rapid classification of tef [Eragrostis tef (Zucc.) Trotter] grain varieties using digital images in combination with multivariate technique. Smart Agric. Technol. 3, 100097. doi: 10.1016/j.atech.2022.100097 38826717

[B10] Bagherpour H. (2026). Machine learning approach for wheat variety identification using single-seed imaging. Sci. Rep. 16 (1), 1–13. doi: 10.1038/s41598-026-35252-8 41605999 PMC12910013

[B11] Barbedo J. G. A. (2018). Factors influencing the use of deep learning for plant disease recognition. Biosyst. Eng. 172, 84–91. doi: 10.1016/j.biosystemseng.2018.05.013 38826717

[B12] Booth R. H. Wholey D. W. (1978). Cassava processing in southeast asia. In Cassava Harvesting and Processing (Ottawa, Canada: International Development Research Centre (IDRC)). pp. 7–11. doi: 10.1093/acprof:oso/9780198737407.003.0006

[B13] Button P. (2006). “ New developments in the international union for the protection of new varieties of plants (UPOV)”, in: XXII International Eucarpia symposium, Section Ornamentals, Breeding for Beauty, 714, 195–210.

[B14] Ceballos H. Iglesias C. A. Pérez J. C. Dixon A. G. O. (2004). Cassava breeding: opportunities and challenges. Plant Mol. Biol. 56, 503–516. doi: 10.1007/s11103-004-5010-5 15630615

[B15] Ding J. Hu J. Lin J. Zhang X. (2024). Lightweight enhanced YOLOv8n underwater object detection network for low light environments. Sci. Rep. 14, 1–22. doi: 10.1038/s41598-024-79211-7 39537721 PMC11561321

[B16] Ding Z. Fu L. Chen G. Yan Y. Tie W. Meng X. . (2025). Coordinated transcriptional regulation of carbohydrate-related pathways contributes to the difference of starch accumulation between starchy cassava and sugary cassava. Carbohydr. Polym. 354, 123314. doi: 10.1016/j.carbpol.2025.123314 39978918

[B17] Douthwaite B. (2020). Development of a cassava seed certification system in Tanzania: Evaluation of CGIAR contributions to a policy outcome trajectory. Available online at: https://hdl.handle.net/10568/110093 (Accessed August 12, 2026).

[B18] Du J. Lu X. Fan J. Qin Y. Yang X. Guo X. (2020). Image-based high-throughput detection and phenotype evaluation method for multiple lettuce varieties. Front. Plant Sci. 11, 563386. doi: 10.3389/fpls.2020.563386 33123178 PMC7573370

[B19] Du R. Shi W. Lu X. Xiang Y. Zhang Y. Feng X. (2026). TrSC2Y: a transfer-learning-based model from UAV hyper-spectra imagery for field-scale canola yield prediction by integrating DSSAT with PROSAIL. Eur. J. Agron. 174, 127926. doi: 10.1016/j.eja.2025.127926 38826717

[B20] Fávero L. P. Belfiore P. Santos H. P. dos Santos M. de Araújo Costa I. P. Junior W. T. (2022). Classification performance evaluation from multilevel logistic and support vector machine algorithms through simulated data in Python. Proc. Comput. Sci. 214, 511–519. doi: 10.1016/j.procs.2022.11.206 38826717

[B21] Gomez D. Selvaraj M. G. Casas J. Mathiyazhagan K. Rodriguez M. Assefa T. . (2024). Advancing common bean (Phaseolus vulgaris L.) disease detection with YOLO driven deep learning to enhance agricultural AI. Sci. Rep. 14, 15596. doi: 10.1038/s41598-024-66281-w 38971939 PMC11227504

[B22] Ha C. D. Hien N. T. Thu P. T. L. (2016). Morphological characterization and classification of cassava (Manihot esculenta Crantz) in Vietnam. Academia J. Biol. 3, 344–351. doi: 10.15625/0866-7160/v38n3.8570

[B23] He P. Chen W. Pang L. Zhang W. Wang Y. Huang W. (2024). “ The survey of one-stage anchor-free real-time object detection algorithms”, in: Sixth Conference on Frontiers in Optical Imaging and Technology: Imaging Detection and Target Recognition, 13156. 1315602. doi: 10.1117/12.3012931

[B24] Helaly A. Mady E. Craker L. (2020). Identification of squash landraces using UPOV descriptors and morphological traits. Al-Azhar J. Agric. Res. 45, 101–113. doi: 10.21608/ajar.2020.149437

[B25] Hershey C. H. (1993). “ Cassava: manihot esculenta crantz,” in Genetic Improvement of Vegetable Crops (Oxford, UK: Pergamon), 669–691. doi: 10.1016/B978-0-08-040826-2.50050-3

[B26] Howeler R. (2004). Cassava in asia:Present situation and its future potential in agro-industry. 189 (17,570), 10–76. doi: 10.1201/9780429035258-17

[B27] Hussain M. (2023). YOLO-v1 to YOLO-v8, the rise of YOLO and its complementary nature toward digital manufacturing and industrial defect detection. Machines 11, 677. doi: 10.3390/machines11070677 30654563

[B28] Jackulin C. Murugavalli S. (2022). A comprehensive review on detection of plant disease using machine learning and deep learning approaches. Measurement: Sensors 24, 100441. doi: 10.1016/j.measen.2022.100441 38826717

[B29] James K. Bradshaw K. (2020). Detecting plant species in the field with deep learning and drone technology. Methods Ecol. Evol. 11, 1509–1519. doi: 10.1111/2041-210X.13473 40046247

[B30] Jung M. Song J. S. Hong S. Kim S. W. Go S. Lim Y. P. . (2021). Deep learning algorithms correctly classify Brassica rapa varieties using digital images. Front. Plant Sci. 12, 738685. doi: 10.3389/fpls.2021.738685 34659305 PMC8511822

[B31] Kai P. M. de Oliveira B. M. da Costa R. M. (2022). Deep learning-based method for classification of sugarcane varieties. Agronomy 12, 2722. doi: 10.3390/agronomy12112722 30654563

[B32] Khan A. T. Jensen S. M. Khan A. R. (2025). Advancing precision agriculture: a comparative analysis of YOLOv8 for multi-class weed detection in cotton cultivation. Artif. Intell. Agric. 15, 182–191. doi: 10.1016/j.aiia.2025.01.013 38826717

[B33] Krochmal A. (1969). Propagation of cassava. World Crops. 21 (3),193–195.

[B34] Kumar S. S. Prasad S. Wani O. A. El-Hendawy S. Mattar M. A. Salem A. (2025). Genetic diversity and agro-morphological characterization of cassava varieties provides insight for breeding and crop improvement. Sci. Rep. 15, 17498. doi: 10.1038/s41598-025-02527-5 40394140 PMC12092572

[B35] Laabassi K. Belarbi M. A. Mahmoudi S. Mahmoudi S. A. Ferhat K. (2021). Wheat varieties identification based on a deep learning approach. J. Saudi Soc Agric. Sci. 20, 281–289. doi: 10.1016/j.jssas.2021.02.008 38826717

[B36] Lange D. M. Przybył K. Łukomski M. Koszela K. Boniecki P. (2018). Neural identification of images showing selected varieties of stored potatoes. J. Res. Appl. Agric. Eng. 63, 110–113. Available online at: https://www.jraae.com/pdf-197956-118195?filename=Neural-identification-of-.pdf (August 12, 2026)

[B37] LeCun Y. Bengio Y. Hinton G. (2015). Deep learning. Nature 521, 436–444. doi: 10.1038/nature14539 26017442

[B38] Legg J. P. Diebiru-Ojo E. Eagle D. Friedmann M. Kanju E. Kapinga R. . (2022). “ Commercially sustainable cassava seed systems in Africa,” in In Root, Tuber and Banana Food System Innovations: Value Creation for Inclusive Outcomes ( Springer International Publishing, Cham), 453–482. doi: 10.1007/978-3-030-92022-7_15

[B39] Mercurio D. I. Hernandez A. A. (2019). “ Classification of sweet potato variety using convolutional neural network”, in: 2019 IEEE 9th international conference on system engineering and technology (ICSET) (Shah Alam, Malaysia: IEEE), 120–125.

[B40] Mohanty S. P. Hughes D. P. Salathé M. (2016). Using deep learning for image-based plant disease detection. Front. Plant Sci. 7, 1419. doi: 10.3389/fpls.2016.01419 27713752 PMC5032846

[B41] Mrisho L. M. Mbilinyi N. A. Ndalahwa M. Ramcharan A. M. Kehs A. K. McCloskey P. C. . (2020). Accuracy of a smartphone-based object detection model, PlantVillage Nuru, in identifying the foliar symptoms of the viral diseases of cassava–CMD and CBSD. Front. Plant Sci. 11, 590889. doi: 10.3389/fpls.2020.590889 33391304 PMC7775399

[B42] Msami J. A. Ndalahwa M. Nickas J. Arbogast M. Nkwabi J. Mwakyusa N. . (2025). Accelerated cassava varietal turnover in Tanzania, a direct result of cassava seed system interventions. Front. Sustain. Food Syst. 9, 1564907. doi: 10.3389/fsufs.2025.1564907

[B43] Murphy K. M. Ludwig E. Gutierrez J. Gehan M. A. (2024). Deep learning in image-based plant phenotyping. Annu. Rev. Plant Biol. 75, 771–795. doi: 10.1146/annurev-arplant-070523-042828 38382904

[B44] Palwankar T. Kothari K. (2022). Real time object detection using ssd and mobilenet. Int. J. For. Res. Appl. Sci. Eng. Technol. 10, 831–834. doi: 10.22214/ijraset.2022.40755

[B45] Plested J. Phiri M. Gedeon T. (2026). Deep transfer learning for image classification: a survey. Artif. Intell. Rev. 49 (3), 100 doi: 10.1007/s10462-026-11491-z 30311153

[B46] Pound M. P. Atkinson J. A. Townsend A. J. Wilson M. H. Griffiths M. Jackson A. S. . (2017). Deep machine learning provides state-of-the-art performance in image-based plant phenotyping. GigaScience 6, 1–10. doi: 10.1093/gigascience/gix083 29020747 PMC5632296

[B47] Ramcharan A. Baranowski K. McCloskey P. Ahmed B. Legg J. Hughes D. P. (2017). Deep learning for image-based cassava disease detection. Front. Plant Sci. 8, 1852. doi: 10.3389/fpls.2017.01852 29163582 PMC5663696

[B48] Ramcharan A. McCloskey P. Baranowski K. Mbilinyi N. Mrisho L. Ndalahwa M. . (2019). A mobile-based deep learning model for cassava disease diagnosis. Front. Plant Sci. 10, 272. doi: 10.3389/fpls.2019.00272 30949185 PMC6436463

[B49] Renjun X. Junliang Y. Yi W. Mengcheng S. (2022). Fault detection method based on improved Faster R-CNN: take ResNet-50 as an example. Geofluids 2022, 7812410. doi: 10.1155/2022/7812410

[B50] Roca W. M. Rodriguez A. Patena L. F. Barba R. C. Toro A. J. (1981). Improvement of a propagation technique for cassava using single leaf-bud cuttings: a preliminary report. Cassava Newslett. 8, 4–5.

[B51] Scaria S. S. Balasubramanian B. Meyyazhagan A. Gangwar J. Jaison J. P. Kurian J. T. . (2024). Cassava (Manihot esculenta Crantz)—a potential source of phytochemicals, food, and nutrition—an updated review. EFood 5, e127. doi: 10.1002/efd2.127 41531421

[B52] Semman A. N. Tesfaye Abebe A. Mulualem Beyene T. Yusuf R. I. Gebresillassie Abtew W. (2025). Characterization of cassava (Manihot esculenta [Cranz]) genotypes sourced from different regions in southwestern Ethiopia using morphological traits. Genet. Resour. Crop Evol. 72, 703–718. doi: 10.1007/s10722-025-02346-7 30311153

[B53] Sheikh M. Iqra F. Ambreen H. Pravin K. A. Ikra M. Chung Y. S. (2024). Integrating artificial intelligence and high-throughput phenotyping for crop improvement. J. Integr. Agric. 23, 1787–1802. doi: 10.1016/j.jia.2023.10.019 38826717

[B54] Shynybay Z. Georgieva T. Nedelcheva E. Alikhanov J. Moldazhanov A. Zinchenko D. . (2026). Comparative study of the performance of SqueezeNet and GoogLeNet CNN models in the identification of Kazakhstani potato varieties. AgriEngineering 8, 17. doi: 10.3390/agriengineering8010017 30654563

[B55] Sun Y. Liu Y. Wang G. Zhang H. (2017). Deep learning for plant identification in natural environment. Comput. Intell. Neurosci. 2017, 7361042. doi: 10.1155/2017/7361042 28611840 PMC5458433

[B56] Sun Y. Sun Z. Chen W. (2024). The evolution of object detection methods. Eng. Appl. Artif. Intell. 133, 108458. doi: 10.1016/j.engappai.2024.108458 38826717

[B57] Wei Y. Wen Y. Huang X. Ma P. Wang L. Pan Y. . (2024). The dawn of intelligent technologies in tea industry. Trends Food Sci. Technol. 144, 104337. doi: 10.1016/j.tifs.2024.104337 38826717

[B58] Yang H. Ni J. Gao J. Han Z. Luan T. (2021). A novel method for peanut variety identification and classification by improved VGG16. Sci. Rep. 11, 15756. doi: 10.1038/s41598-021-95240-y 34344983 PMC8333428

[B59] Zhang L. Bai W. Du B. Wang D. Liu Y. Long J. (2025). An insulator defect detection model based on improved YOLOv8. J. Physics: Conf. Ser. 2993, 12059. doi: 10.1088/1742-6596/2993/1/012059

[B60] Zhang P. Li D. (2022). YOLO-VOLO-LS: a novel method for variety identification of early lettuce seedlings. Front. Plant Sci. 13, 806878. doi: 10.3389/fpls.2022.806878 35283870 PMC8909383

[B61] Zhang Z. Raza A. Yang M. Lu Y. Hu Y. (2026). A hybrid and high-performance model for tea cultivars discrimination using canopy images with convolutional neural network and machine learning algorithm. Comput. Electron. Agric. 240, 111140. doi: 10.1016/j.compag.2025.111140 38826717

[B62] Zhu S. Zhang J. Chai M. Xu X. Song P. Zhang J. . (2019). A rapid and highly efficient method for the identification of soybean seed varieties: hyperspectral images combined with transfer learning. Molecules 25, 152. doi: 10.3390/molecules25010152 31905957 PMC6982693

